# Cytotaxonomy of unionid freshwater mussels (Unionoida, Unionidae) from northeastern Thailand with description of a new species

**DOI:** 10.3897/zookeys.514.8977

**Published:** 2015-07-27

**Authors:** Bangon Kongim, Chirasak Sutcharit, Somsak Panha

**Affiliations:** 1 Biodiversity and Traditional Knowledge Research Unit, Department of Biology, Faculty of Science, Mahasarakham University, Maha Sarakham 44150, Thailand; 2 Animal Systematics Research Unit, Department of Biology, Faculty of Science, Chulalongkorn University, Bangkok 10330, Thailand

**Keywords:** Chromosome, mussel, karyotype, systematics, Southeast Asia, cryptic species

## Abstract

Morphological and chromosomal characteristics of a number of unionid freshwater mussels were studied from northeastern Thailand. Karyotypes of eight species from seven genera (*Chamberlainia*, *Ensidens*, *Hyriopsis*, *Physunio*, *Pseudodon*, *Scabies* and *Trapezoideus*) were examined. Six species possess 2*n* = 38 karyotypes, whereas *Scabies crispata* and an unidentified *Scabies* sp. lack three small chromosome pairs, giving a diploid number of 32. Moreover, the karyotypes of the unidentified *Scabies* differ from *Scabies crispata* as it exhibits a telocentric chromosome pair (6m + 7sm + 2st + 1t). Most of the conchological characters also differ between the two species – adult size, colour pattern, muscle scars, pseudocardinal and lateral teeth. The name *Scabies songkramensis* **sp. n.** is proposed for the unidentified species, and its description is included in this paper. Interestingly, seven species contain mostly bi-armed chromosomes, but only the mud-dweller in stagnant water, *Ensidens ingallsianus*, contains predominantly five telocentric pairs. In addition, the marker chromosome characteristics of an unbalanced long arm, twisted centromere, a wider angle 180° arrangement, a twisted arm and telomeric end union reported in this study are described for the first time for unionid mussels.

## Introduction

The Unionidae is numerically the largest family of both extant and extinct freshwater mussels and includes over 670 species worldwide with about 220 species occurring in Indotropica (Graf and Cummings 2007). Such high species diversity and wide distribution make the unionid mussels very attractive for systematic and bio-geographical studies. However, environmental problems, including water pollution, threatens the survival of many species today, and many populations in many parts of the world have been reported as declining (Williams et al. 1993; Vaughn and Tayler 1999; Sethi et al. 2004; Haag and Williams 2014). As a response, taxonomic and systematic studies of unionids that integrate conchological and anatomical analyses with molecular phylogenies have increased over the last two decades.

Most studies have dealt with American, European and Australasian taxa (Rosenberg et al. 1994, 1997; Graf and Ó Foighil 2000; Hoeh et al. 2001; Graf and Cummings 2011; Graf 2013; Lopes-Lima et al. 2014; Prié and Puillandre 2014; Graf et al. 2015), whereas Asian taxa have largely been neglected. The monographs by Haas (1969) and Brandt (1974) have reported taxonomic surveys of Thai species. Nevertheless, recent reassessments by other malacologists have revealed some new and unknown species (Graf 2002; Deein et al. 2003) and there are still many localities that have never been surveyed. Owing to their conservative morphological diversity, it is has not been easy to establish a reliable phylogeny for unionids. Identification of species is often difficult due to morphological variation among individuals and within regional populations, termed ecophenotypic variability (Roe et al. 2001; Plouviez et al. 2009; Vannarattanarat et al. 2014). The plasticity of shell characters is well-known amongst the Unionoida (e.g. Graf 2000; Baker et al. 2004; Marshall et al. 2014). Tests of phylogenetic hypotheses on the basis of other data sources, such as those derived from molecules and chromosomes, are therefore likely to be informative. However, such approaches have as yet been attempted only on a limited number of taxa and there are still very few studies in Asian and African regions (Lopes-Lima et al. 2014; Marshall et al. 2014; Graf et al. 2015).

Several sympatric species have been recorded in numerous Thai localities (Brandt 1974; Panha 1990), raising many interesting taxonomic and ecological questions. Some of these questions have been addressed in a few publications on some biological aspects such as the relationships of mussels and their fish hosts or ‘glochidiosis’ (Panha 1992; 1993a,b). Whilst chromosomal data of some Thai unionids have been described (Meesukko 1996; Deein et al. 2003; see also Table 1), the number of karyotyped species comprise fewer than 30% of the total species diversity in the family.

**Table 1.** Data summary. This table shows the number of specimens examined (No.), locality, diploid number (*2n*), fundamental number (FN), karyotype pattern and chromosome markers of the Unionidae species included the present study. The numbered localities are presented in Figure 1. Abbreviations: m, metacentric; sm, submetacentric; st, subtelocentric; t, telocentric.

Species	No.	Locality	2*n*	FN	Karyotype formula	Marker chromosome (pair number)
**UNIONIDAE**						
**Subfamily Hyriopsinae**						
*Chamberlainia hainesiana*	2	2,3	38	76	4m + 9sm + 6st	5 and 13 unbalance of long arm
*Hyriopsis bialatus*	10	1, 2, 3	38	72	6m + 7sm + 4st + 2t	6 telomeric end union
**Subfamily Parreysiinae**						
*Scabies crispata*	10	1, 2, 3	32	64	6m + 7sm + 3st	10 twisted arm
*Scabies songkramensis* sp. n.	10	4, 5	32	62	6m + 7sm + 2st + 1t	15 small and twisted arm
**Subfamily Pseudodontinae**						
*Pseudodon mouhoti*	6	1, 2, 3	38	74	6m + 6sm + 6st +1t	7 twisted centromere
**Subfamily Rectidentinae**						
*Ensidens ingallsianus*	6	1, 2, 3	38	46	3m + 4sm + 7st + 5t	1 unbalance of long arm 6 and 13 wider angle 180° arrangement and twisted centromere
*Physunio inornatus*	10	1, 2, 3	38	74	3m + 9sm + 6st + 1t	4 wider angle 180° arrangement 8 twisted centromere
*Trapezoideus exolescens*	10	1, 2, 3	38	74	3m + 10sm + 5st + 1t	3 unbalance of long arm

Here we examine the karyotypes of eight species of unionids from northeastern Thai that represent seven genera (and four subfamilies): *Chamberliania*, *Hyriopsis* (Hyriopsinae); *Scabies* (Parreysiinae); *Pseudodon* (Pseudodontinae); *Ensidens*, *Physunio*, *Trapezoideus* (Rectidentinae). All these genera are considered to be completely different from each other on a morphological basis (Brandt 1974; Panha 1990).

## Materials and methods

**Figure 1.**
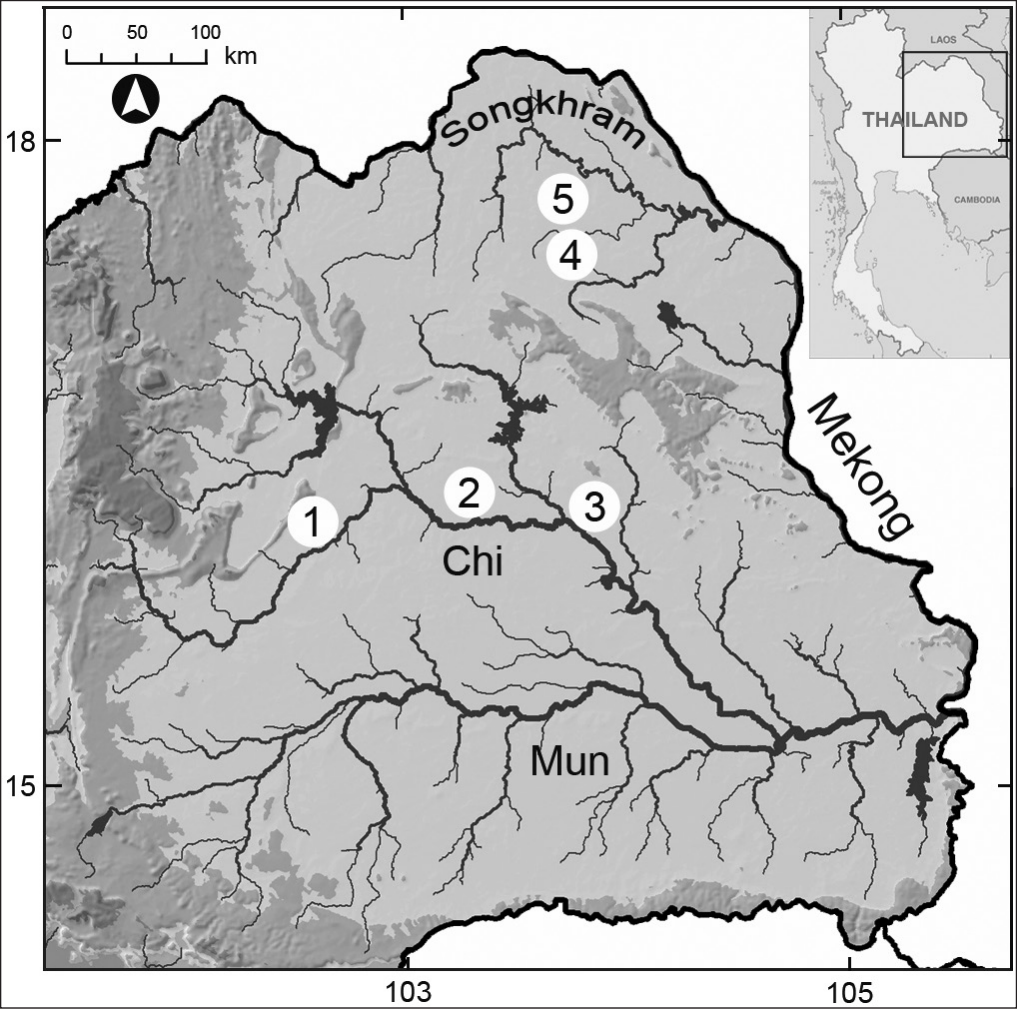
Sampling locations for unionids in northeastern Thailand: **1** Ban Tha Nanglian, Chonnabot, Khon Kaen (16°1'21"N; 102°33'34"E) **2** Ban Tha Khonyang, Kantharawichai, Maha Sarakham (16°14'1"N; 103°16'1"E) **3** Ban Tha Krai, Selaphum, Roi Et (16°2'0"N; 103°56'2"E) **4** Ban Klang Charern, Pangkon, Sakon Nakorn (17°24'22"N; 103°50'1"E) **5** Kamtakla, Sakon Nakorn (17°49'32"N; 103°47'10"E).

**Figure 2.**
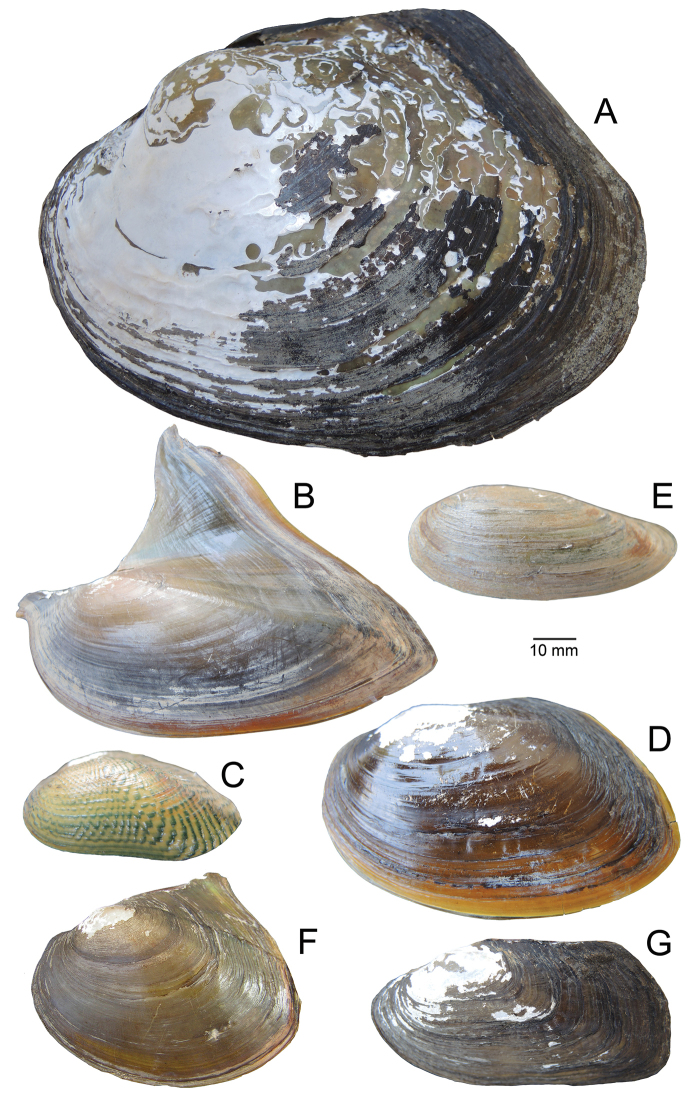
Comparative external views of shell valves of unionids studied: **A** *Chamberlainia hainesiana* **B** *Hyriopsis bialatus* **C** *Scabies crispata* **D** *Pseudodon mouhoti* **E** *Ensidens ingallsianus* **F** *Physunio inornatus* **G** *Trapezoideus exolescens*.

The localities and shell characteristics of each species are given in Figs 1, 2 and Table 1. Species identifications were made using Brant (1974) and Sutcharit et al. (2013). Comparisons with type specimens in the Senckenberg Museum, Frankfurt (SMF) were also conducted. Chromosome preparations were made from gill tissue by the air-drying method, modified from Patterson and Burch (1978), Deein et al. (2003) and Kongim et al. (2006, 2009, 2010). Living animals recently collected from the wild were treated with colchicine solution for 4 h at a final concentration of 0.01 M. Gill filaments were removed, cut into small pieces, and soaked in 0.075 M KCl for 45 min. The cells were then harvested by centrifugation at 1500 rpm. After fixation and rinsing in 3:1 (v/v) methanol: acetic acid, the cell suspension was pipetted onto microscope slides on warm plates (60 °C) and allowed to dry under controlled conditions for optimum spread. Chromosomes were stained with 4% (w/v) Giemsa solution for 10 min. For the karyotype analysis, metaphase plates in which the chromosomes were clearly differentiated within the cells were selected for study. Photomicrographs of 25 well-spread metaphase cells were measured for relative chromosome length and centromeric index. Mitotic karyotypes were arranged and numbered for chromosome pairs in order of decreasing mean relative length. The nomenclature for morphological chromosome types was derived from Levan et al. (1964).

Abbreviations for figures and measurements: aa, anterior adductor; muscle scar; lt, lateral teeth; pa, posterior adductor muscle scar; pl, pallial line; pt, pseudocardinal tooth; H, height of valves; L, length of valves; W, width of valves.

### Institutional abbreviations

CUMZ Chulalongkorn University, Museum of Zoology, Bangkok, Thailand

SMF Forschungsinstitut und Naturmuseum Senckenberg, Frankfurt, Germany

ZMMSU Zoological Museum of Mahasarakham University, Thailand.

## Results

### Karyotype

**Figure 3.**
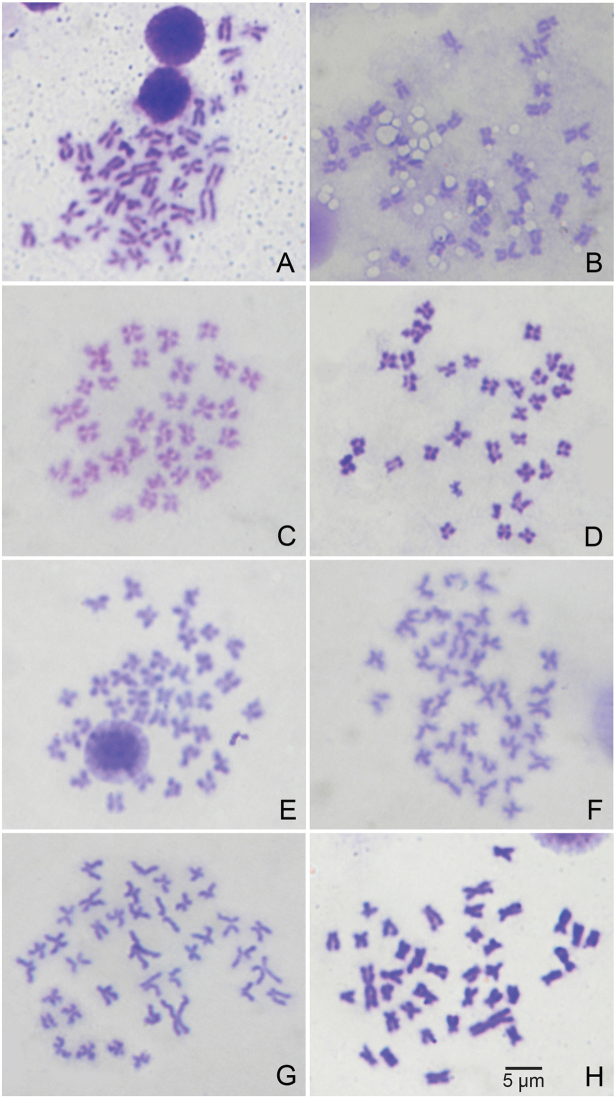
Mitotic chromosomes of unionids studied: **A** *Chamberlainia hainesiana* **B** *Hyriopsis bialatus* **C** *Scabies crispata* **D** *Scabies songkramensis* sp. n. **E** *Pseudodon mouhoti* **F** *Ensidens ingallsianus* **G** *Physunio inornatus* **H** *Trapezoideus exolescens*.

**Figure 4.**
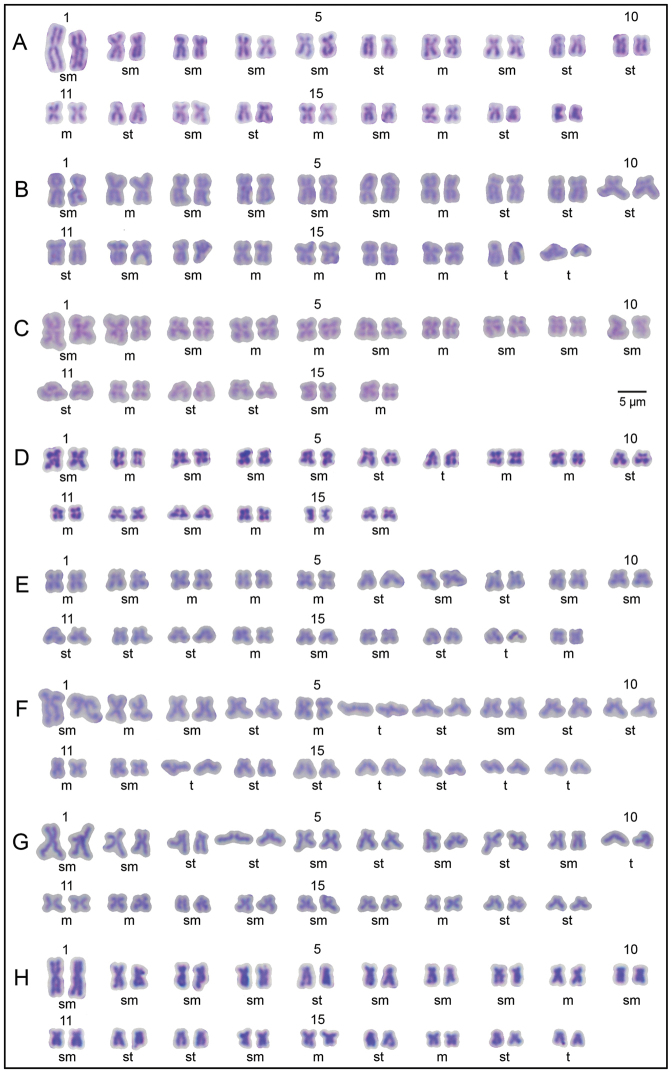
Karyotypes of unionids studied: **A** *Chamberlainia hainesiana* **B** *Hyriopsis bialatus* **C** *Scabies crispata* **D** *Scabies songkramensis* sp. n. **E** *Pseudodon mouhoti* **F** *Ensidens ingallsianus* **G** *Physunio inornatus* **H** *Trapezoideus exolescens*. Abbreviations: m, metacentric; sm, submetacentric; st, subtelocentric; t, telocentric; numbers 1, 5, 10, 11, 15 represent the pair numbers.

The karyotype of six species consists of 2*n* = 38 chromosomes, but two species (*Scabies crispata* and an unidentified *Scabies* sp.) showed 2*n* = 32. In all samples examined, no sex chromosome heteromorphism or secondary constrictions were evident. The fundamental numbers (FN) varied among species, ranging from 46 to 76 (Figs 3, 4 and Table 1). Seven species contain metacentric dominant chromosomes (12–13 pairs), but only *Ensidens ingallsianus* contains 12 pairs of the telocentric dominant category. The two large pearl mussels (*Chamberlainia hainesiana* and *Hyriopsis bialatus*) plus one medium-sized species (*Trapezoideus exolescens*) have the same numbers of metacentric and telocentric chromosomes consisting of 13 + 6 pairs with slightly different arrangements (Table 1). *Chamberlainia hainesiana* possesses the largest chromosome pair 1, and has unbalanced arms on chromosome pairs 5 and 13. *Hyriopsis bialatus* possesses distinct chromosome markers in having a short arm pair 6 with a telomere end union.

The karyotype of *Scabies crispata* is almost identical to that of *Scabies songkramensis* sp. n., but the latter differs in having a telocentric pair 7. The FN values were dissimilar at 64 and 62, respectively (Figs 3, 4 and Table 1). Both species show chromosome markers of a twisted arm on chromosome pair 10 and 15, respectively.

The karyotypes of *Pseudodon mouhoti* consists of 6m + 6sm + 6st + 1t with twisted centromere pair 7. The three members of the subfamily Rectidentinae (i.e. *Ensidens ingallsianus*, *Physunio inornatus* and *Trapezoideus exolescens*) are different from each other in FN value, size arrangement and morphology of chromosomes (Table 1), but all three exhibit the largest chromosome pair 1. *Ensidens ingallsianus* distinct chromosome markers of having long arm characters of the first pairs, with the non-identical left and right long arms, as well as exhibiting a remarkably wide angle (about 180°) arrangement of chromosome pairs 6 and 13. *Physunio inornatus* also exhibits a slightly smaller angle at 100° in pair 4, and pair 8 shows a twisted centromere. The distinct chromosome markers in *Trapezoideus exolescens* are the non-identical left and right long arms in pair 3 (Table 1).

## Systematics

### Family Unionidae Rafinesque, 1820

#### Genus Scabies

Taxon classificationAnimaliaUnionoidaUnionidae

Haas, 1911

##### Type species

(by subsequent designation of Haas 1969: 63) *Unio scobinatus* Lea, 1856. Recent, Southeast Asia. Gender masculine.

#### Scabies songkramensis

Taxon classificationAnimaliaUnionoidaUnionidae

Kongim & Panha sp. n.

http://zoobank.org/C55BB4DA-BACA-40A6-AF97-8496C3B2FC14

**Figure 5.**
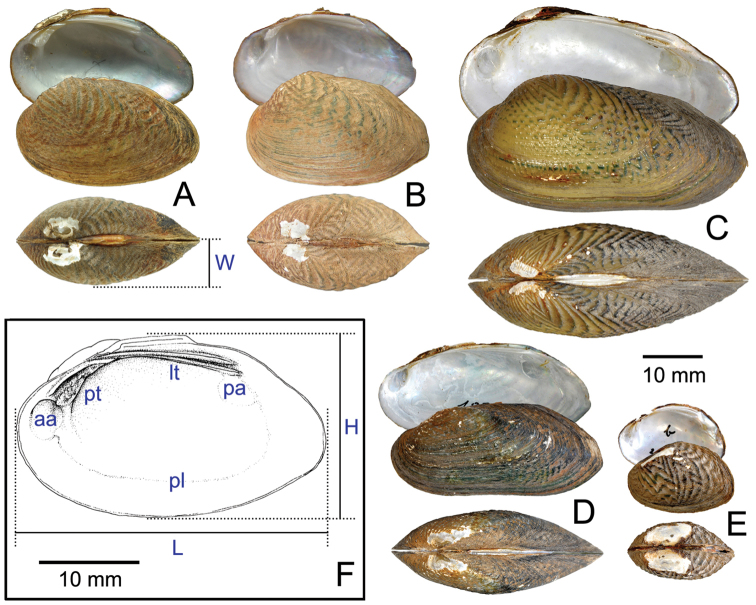
Shell valves of **A, B** *Scabies songkramensis* sp. n., **A** holotype ZMMSU 00500 and **B** paratype ZMMSU 00501. **C** *Scabies crispata*, Brandt collection SMF 188682 from Bangkok, Thailand **D** *Scabies nucleus* Brandt collection SMF 198394 from Mekong River, Pakse, Laos **E** *Scabies phaselus* Brandt collection SMF 188695 from Takrong River, Nakon Ratchsrima, and **F** hinge plates of *Scabies songkramensis* sp. n., holotype, with illustrating and measurements terminology. Abbreviations: aa, anterior adductor muscle scar; lt, lateral teeth; pa, posterior adductor muscle scar; pl, pallial line; pt, pseudocardinal tooth; H, height of valves; L, length of valves; and W, width of valves.

Fig. 5A, B, F ; Table 3

##### Type material.

Holotype ZMMSU 00500 (length 30 mm, height 18 mm, width 7.5 mm) Paratypes: ZMMSU 00501 (20 shells; length 29–33 mm, height 17–19 mm, width 7–8 mm); CUMZ (five shells).

##### Type locality.

Houy Plahang stream in Songkram River Basin, Ban Klang Charern, Pangkon, Sakon Nakorn, Thailand – 17°24'22"N, 103°50'1"E. Type locality indicated in Fig. 1, locality 4).

##### Etymology.

The specific name *songkramensis* refers to the Songkram River, type locality of this new species. Authorship of this new species is to be credited to Kongim and Panha in Kongim, Sutcharit and Panha.

##### Description.

Shell of medium size (length 29–33 mm), ovate in outline, H/L ratio = 0.59, anterior portion rounded, umbonal area elevated and sloping downwards posteriorly. Underlying shell colour brown. Shell sculptured with a series of coarse, v-shaped ribs radiating outwards from umbo; v-line arrangement loose, with 4-fold number on 10 mm; posterior slope with coarse and distinct ridges. Sculpture reduced to nearly obsolete near ventral and posterior shell margin. Periostracum brown, tending towards dark green where ribs are worn. Hinge plate well-developed; pseudocardinal tooth (pt) forming a thickened plate and raised lamelliform on right valve, but thinner and also raised lamelliform on left valve. Two well-developed posterior lateral teeth (lt) present in each valve, long and sharp. Anterior adductor muscle scar (aa) prominent and deeply impressed; posterior adductor muscle scar (pa) shallow; pallial line (pl) faintly impressed. Nacre bluish-white with little iridescence.

##### Remarks.

The new species differs from the closely related *Scabies crispata* (Gould) and *Scabies phaselus* (Lea) by having a smaller, harder, thicker, ovate shell that is brown in colour, with dark brown v-line sculpture. The two other species have larger, more elongate shells that are yellowish brown in colour, combined with greenish v-line sculpturing in *Scabies crispata* and a nearly smooth shell surface in *Scabies phaselus*. *Scabies songkramensis* sp. n. differs from *Scabies nucleus* (Lea) in having a larger shell and v-line sculpture, compared with w-line sculpture in *Scabies nucleus*.

##### Habitat.

*Scabies songkramensis* sp. n. occurs in a small tributary of the Songkhram River. It lives in shallow water in a sandy-gravel substrate, or less frequently in sandy-mud. This new species is currently known only from the type locality, approximately 100 km from the main stem of the Songkhram River (Fig. 1, locality 4), in slow moving water at depths that ranged from 0.5 to 2 m in the wet season (i.e. from June to October).

## Discussion

The diploid numbers of six species in the three subfamilies, Hyriopsinae, Pseudodontinae and Rectidentinae, showed the same chromosome number (2*n* = 38), which is similar to unionid taxa in other regions (Vitturi et al. 1982; Meesukko 1996; Jenkinson 2014; see also Table 2). An investigation of two species of *Alasmidonta* and four species of *Anodonta* also showed a similar chromosome number (2*n* = 38) and fundamental arm number, FN = 76 (see Table 2). In other regions, the Parreysiinae is traditionally considered as more primitive than other subfamilies (Bieler et al. 2010; Carter et al. 2011; Whelan et al. 2011; Graf 2013). However, our data showed that *Scabies crispata* and *Scabies songkramensis* sp. n. (Parreysiinae) had the lowest diploid number among the Unionidae (2*n* = 32), which is the same as *Elliptio complanata* (Table 2, Lillie 1901), although Park and Burch (1995) reported the chromosome number of *Elliptio complanata* from Ocqueoc River, Michigan, USA, as being 2*n* = 38. This case should be re-evaluated carefully, especially in terms of the species identification. Unfortunately, we cannot clarify the taxonomic status of the previous *Elliptio complanata* to determine this variation in the chromosome number.

**Table 2.** The diploid (*2n*), haploid (*n*) and fundamental number (FN) for the Unionoida. Data for the Unionidae plus three additional families (Hyriidae, Mutelidae and Margaritiferidae) are included in the table. References as follows: (1) Lillie (1901); (2) McMichael and Hiscock (1958); (3) Griethuysen et al. (1969); (4) Nadamitsu and Kanai (1975); (5) Jenkinson (1976); (6) Vitturi et al. (1982); (7) Park and Burch (1995); (8) Ebied (1998); (9) Jara-Seguel et al. (2000); (10) Wang et al. (2000); (11) Shan et al. (2001) (12) Deein et al. (2003); (13) Woznicki (2004); (14) Woznicki and Jankun (2004) and (15) Carrilho et al. (2008). Abbreviations: m, metacentric; sm, submetacentric; st, subtelocentric; t, telocentric; a, acrocentric.

Species	2*n*	*n*	FN	Karyotype	Locality	References
Family Hyriidae						
*Diplodon chilensis*	34	–	–	9m + 8sm	Chile	9
Family Mutelidae						
*Alathyria pertexta*	34	–	–	–	Australia	2
*Mutela rostrata*	20	–	–	2m + 2sm + 6a	Egypt	8
*Velesunio ambiguus*	34	–	–	–	Australia	2
*Velesunio legrandi*	34	–	–	–	Tazmania	2
Family Margaritiferidae						
*Margaritifera margaritifera*	38	–	–	–	USA	5
*Margaritifera laevis*	38	19	76	19sm	Japan	4
Family Unionidae						
*Alasmidonta arcula*	38	–	–	–	USA	5
*Alasmidonta marginata*	38	–	–	10m + 7sm + 2sm	USA	5
*Anodonta anatina*	38	–	76	10m + 3s/m + 6sm	Netherlands	3
*Anodonta anatina*	38	–	76	6m + 12sm + 1st	Poland	14
*Anodonta cygnea*	38	–	76	6m + 12sm + 1st	Portugal	15
*Anodonta grandis*	38	–	–	6m + 12sm + 1st	USA	5
*Anodonta woodiana*	38	–	76	–	Poland	13
*Anodonta woodiana woodiana*	38	–	76	–	China	11
*Anodontoides ferussacianus*	38	–	–	9m + 10sm	USA	5
*Elliptio complanata*	–	16	–	–	USA	1
*Elliptio complanata*	38	–	–	–	USA	7
*Gonidea angulata*	38	–	–	–	USA	5
*Hyriopsis cumingii*	38	–	–	–	China	10
*Inversidens japonensis*	38	–	76	6m + 13sm	Japan	4
*Lampsillis radiate luteola*	38	–	–	–	USA	5
*Lasmigona costata*	38	–	–	9m + 7sm + 3st	USA	5
*Potamilus alatus*	38	–	–	–	USA	5
*Pseudodon obovalis omiensis*	38	–	76	9m + 10sm	Japan	4
*Ptychobranchus fasciolaris*	38	–	–	8m + 10sm + 1st	USA	5
*Quadrula quadrula*	38	–	–	–	USA	5
*Solenaia khwaenoiensis*	37	19	–	3m + 15sm + 1st	Thailand	12
*Toxolasma lividus grans*	38	–	–	–	USA	5
*Tritigonia verrucosa*	38	–	–	–	USA	5
*Unio elongatulus*	28	–	–	10m + 4sm	Egypt	8
*Unio elongatulus*	–	19	–	–	Italy	6
*Unio pictorum*	38	–	76	8m + 1m/sm + 10sm	Netherlands	3
*Villosa iris*	38	–	–	11m + 6sm + 2st	USA	5
*Villosa lienosa*	38	–	–	–	USA	5

**Table 3.** Comparisons of shell characteristics of the new species compared with those of the three other Thai species of *Scabies*.

Characteristics	*Scabies songkramensis* sp. n.	*Scabies crispata*	*Scabies nucleus*	*Scabies phaselus*
Length of valves; L (mm)	29–33 29.60 ± 0.57	30–39 35.40 ± 2.33	16–19 18.00 ± 0.40	30–35 32.60 ± 1.85
Height of valves; H (mm)	17–19 17.60 ± 0.57	14–17 15.88 ± 0.68	11–13 12.20 ± 0.67	13–17 15.26 ± 0.55
Width of valves; W (mm)	7–8 7.51 ± 0.35	5.5–7.5 6.57 ± 0.42	3.5–4.5 4.23 ± 0.87	5.5–7 5.95 ± 0.39
H/L ratio	0.59 ± 0.01	0.46 ± 0.31	0.71 ± 0.01	0.48 ± 0.32
Shell shape	Ovate	Elongate cuneiform	Subquadrate	Elongate with ventral margin concave
Shell colour	Greenish brown	Dark greenish	Greenish	Dark greenish
Shell sculpture	Coarse, obtuse	Fine, glossy	Coarse, obtuse	Fine, glossy
Line of shell sculpture	Loose, distinct v-line	v or w-line	v-line	Dense, wavy line
Fold number on shell sculpture on 10 mm	4	6	6	9
Shell thickness	Thick	Thin	Thick	Thin
Nacre colour	Bluish-white	Milky-white	Bluish-white	Milky-white
Pseudocardinals tooth	Thick plate	Large, deep fracture	Thick, stumpy, short, deep fracture	Large, short, triangular, pointed crest
Muscle scars	Deep and narrow in anterior, shallow in posterior	Deep in anterior, very shallow in posterior	Distinct, deep in anterior	Deep in anterior

McMichael and Hiscock (1958) identified 2*n* = 34 as the chromosome number for three species of Mutelidae and 1 species of Hyriidae, the latter a more primitive family than the Unionidae. However, chromosome number has been, so far, of little used for the taxonomy of unionid mussels. The other recent reports of different diploid number are from *Solenaia khwaenoiensis* from Thailand with the unusual 37 (2*n*) chromosomes (Deein et al. 2003) and from *Unio elongatulus* from Egypt with 28 (2*n*) (Ebied 1998). However, *Unio elongatulus* was previously karyotyped from Italy and this *Unio* species has only been described from the upper Nile in Ethiopia, whereas these were caught in the lower part of this river in Egypt. This misidentification was made probably with one of the common genus *Coelatura* in the lower Nile River. Interestingly *Coelatura* also belongs within Parreysiinae. The karyotype of most species has not been studied in detail and additional characters might be useful for identification to species level. This study revealed that the Parreysiinae genus *Scabies*, which possesses a lower chromosome number than others of its subfamily, is significant because it has not been reported previously.

The karyotypes of all eight species of unionids studied here differ in the degree of asymmetry (sub-telocentric and telocentric). Primitive karyotypes typically exhibit low asymmetry and derived karyotypes show higher asymmetry (Diupotex-Chong et al. 2004; Kongim et al. 2010). Thus, the karyotype of *Scabies crispata* is assumed to exhibit a primitive character among Southeast Asian unionids, whereas the karyotype with the highest asymmetry was exhibited by *Ensidens ingallsianus* (Rectidentinae), which is assumed to be a derived form.

Marker chromosomes such as telomeric end union, wider angle arrangement and others, are useful in taxonomy and systematics (Gomes et al. 2011). Our data show that marker chromosome arrangement varies among species and so may have diagnostic significance. The unbalance of the long arm and the twisted centromere are found in most cases in four chromosome pairs in three species. The latter wider angle 180° arrangement, and twisted arm are found in two chromosome pairs in two species. The last telomeric end union is found in only one pair of a single species (*Hyriopsis bialatus*) and that could be a diagnostic feature for this species. All of the marker chromosomes are different in their chromosome structure, especially the telomeric end union, whereby the sticky end in the telomere of the two chromatids cause the fusing together that is the telomeric arrangement. Overall, the data indicated that several chromosomal re-arrangements seem to have taken place during the karyo-evolutionary history of unionid species, mainly driven by reciprocal translocation (Halnan 1989; Rooney and Czepulkowski 1992; Clark and Wall 1996; Rickart et al. 1999). This karyological differentiation is not only related to geographical isolation, but it also indicates reproductive incompatibility and the occurrence of different evolutionary mechanisms of translocation. This karyological evidence was supported by the differences in their morphology and geographic separation. The Parreysiinae has been reported to be an early branch from the common ancestor leading to the other subfamilies with the other subfamilies being proposed as sister groups (Whelan et al. 2011; Graft 2013). Differences in chromosome number may be an isolation mechanism in each subfamily, as supported by the molecular phylogenetic tree of freshwater mussels (Bieler et al. 2010; Carter et al. 2011; Whelan et al. 2011; Graf and Cummings 2011; Graf 2013).

The karyotype is generally a species-specific character, and as such is useful in species discrimination (White 1978; Halnan 1989; King 1993; Clark and Wall 1996; Kolnicki 2000). Karyological data have been used for species-level classification in several molluscan groups, including *Atlanta*, *Bellamya*, *Goniobasis* and *Viviparus* (Zhou et al. 1988; Dillon 1991; Thiriot-Quièvreux and Seapy 1997; Baršienė et al. 2000). Chromosome variations, in terms of both the number, karyotype pattern, and the marker chromosome, have been implicated as a primary isolating mechanism for speciation in the polymorphic *Sphaerium corneum* (see Petkevičiūtė et al. 2006). Therefore, cytogenetic study is an efficient tool for systematic approaches (cytotaxonomy) in several molluscan groups, where it is helpful in discriminating between morphologically similar species (cryptic species), since the karyotype itself probably represents a character that is resistant to environmental, behavioural or physiological influences (White 1978; Baršienė 1994; Aldridge 2000; Bauer 2001; Sumner 2003).

## Supplementary Material

XML Treatment for Scabies

XML Treatment for Scabies songkramensis

## References

[B1] Aldridge DC (2000) The impacts of dredging and weed cutting on a population of freshwater mussels (Bivalvia: Unionidae). Biological Conservation 95: 247–257. doi: 10.1016/S0006-3207(00)00045-8

[B2] Baker AM Sheldon F Somerville J Walker KF Hughes JM (2004) Mitochondrial DNA phylogenetic structuring suggests similarity between two morphologically plastic genera of Australian freshwater mussels (Unionoida, Hyriidae). Molecular Phylogenetics and Evolution 32: 902–912. doi: 10.1016/j.ympev.2004.02.017 15288065 10.1016/j.ympev.2004.02.017

[B3] Baršienė J (1994) Chromosome set changes in molluscs from highly polluted habitats. In: Beaumont AR (Ed.) Genetics and Evolution of Aquatic Organisms. Chapman and Hall, London, 434–447.

[B4] Baršienė J Ribi G Barsyte D (2000) Comparative karyological analysis of five species of *Viviparus* (Gastropoda: Prosobranchia). Journal of Molluscan Studies 66: 259–271. doi: 10.1093/mollus/66.2.259

[B5] Bauer G (2001) Framework and driving forces for the evolution of naiad life histories. In: Bauer G Wächtler K (Eds) Ecology and Evolution of the Freshwater Mussels Unionoida. Springer Verlag, Berlin, 234–255. doi: 10.1007/978-3-642-56869-5_13

[B6] Bieler R Carter JG Coan EV (2010) Classification of Bivalve Families. In: Bouchet P Rocroi JP (2010) Nomenclator of Bivalve Families Malacologia 52(2): 113–133. doi: 10.4002/040.052.0201

[B7] Brandt RAM (1974) The non marine aquatic Mollusca of Thailand. Archiv für Molluskenkunde 105: 1–423.

[B8] Carrilho J Leitão A Vicente C Malheiro I (2008) Cytogenetics of *Anodonta cygnea* (Mollusca: Bivalvia) as possible indicator of environmental adversity. Estuarine, Coastal and Shelf Science 80: 303–306. doi: 10.1016/j.ecss.2008.07.019

[B9] Carter JG Altaba CR Campbell DC Harries PJ Skelton P (2011) A synoptical classification of the Bivalvia (Mollusca). Paleontological Contributions of the Paleontological Institute, University of Kansas 4: 1–47.

[B10] Clark MS Wall WJ (1996) Chromosome: The Complex Code. Alden Press, Oxford, 345 pp. doi: 10.1007/978-94-009-0073-8

[B11] Deein G Unakornsawat Y Rattanadaend P Sutcharit C Kongim B Panha S (2003) A new species of *Solenaia* from Thailand (Bivalve: Unionidae: Ambleminae). The Natural History Journal of Chulalongkorn University 3: 53–58.

[B12] Dillon RT (1991) Karyotypic evolution in pleurocerid snails. II. *Pleurocera*, *Goniobasis* and *Juga*. Malacologia 33: 339–344.

[B13] Diupotex-Chong ML Cazzaniga N Hernández-Santoyo A Betancourt-Rule JM (2004) Karyotype description of *Pomacea patula catemacensis* (Caenogastropoda, Ampullariidae), with an assessment of the taxonomic status of *Pomacea patula*. Biocell 28: 279–285. 15633451

[B14] Ebied ABM (1998) Karyological studies on three Egyptian freshwater species of order Eulamellibranchiata (Bivalvia-Mollusca). Cytologia 63: 17–26. doi: 10.1508/cytologia.63.17

[B15] Gomes NM Ryder OA Houck ML Charter SJ Walker W Forsyth NR Austad SN Venditti C Pagel M Shay JW Wright WE (2011) Comparative biology of mammalian telomeres: hypotheses on ancestral states and the role of telomeres in longevity determination. Aging Cell 10: 761–768. doi: 10.1111/j.1474-9726.2011.00718.x 21518243 10.1111/j.1474-9726.2011.00718.x PMC3387546

[B16] Graf DL (2000) The Etherioidea revisited: a phylogenetic analysis of hyriid relationships (Mollusca: Bivalvia: Paleoheterodonta: Unionoida). Occasional Papers of the University of Michigan Museum of Zoology 729: 1–21.

[B17] Graf DL (2002) Molecular phylogenetic analysis of two problematic freshwater genera (*Unio* and *Gonidea*) and a re-evaluation of the classification of Nearctic Unionidae (Bivalvia: Palaeoheterodonta: Unionoida). Journal of Molluscan Studies 68: 65–71. doi: 10.1093/mollus/68.1.65

[B18] Graf DL (2013) Patterns of freshwater bivalve global diversity and the state of phylogenetic studies on the Unionoida, Sphaeriidae, and Cyrenidae. American Malacological Bulletin 31: 135–153. doi: 10.4003/006.031.0106

[B19] Graf DL Cummings KS (2007) Review of the systematics and global diversity of freshwater mussel species (Bivalvia: Unionoida). Journal of Molluscan Studies 73: 291–314. doi: 10.1093/mollus/eym029

[B20] Graf DL Cummings KS (2011) Freshwater mussel (Mollusca: Bivalvia: Unionoida) richness and endemism in the ecoregions of Africa and Madagascar based on comprehensive museum sampling. Hydrobiologia 678: 17–36. doi: 10.1007/s10750-011-0810-5

[B21] Graf DL Ó Foighil D (2000) The evolution of brooding characters among the freshwater pearly mussels (Mollusca: Bivalavia: Unionoidea) of North America. Journal of Molluscan Studies 66: 157–170. doi: 10.1093/mollus/66.2.157

[B22] Graf DL Jones H Geneva AJ Pfeiffer JM Klunzinger MW (2015) Molecular phylogenetic analysis supports a Gonwanan origin of the Hyriidae (Mollusca: Bivalvia: Unionida) and the paraphyly of Australasian taxa. Molecular Phylogenetics and Evolution 85: 1–9. doi: 10.1016/j.ympev.2015.01.012 25659337 10.1016/j.ympev.2015.01.012

[B23] Griethuysen GA van Kiauta B Butot LJM (1969) The chromosomes of *Anodonta anatina* (Linnaeus, 1758) and *Unio pictorum* (Linnaeus, 1758) (Mollusca: Bivalvia: Unionidae). Basteria 33: 51–56.

[B24] Haag WR Williams JD (2014) Biodiversity on the brink: an assessment of conservation strategies for North American freshwater mussels. Hydrobiologia 735: 45–60. doi: 10.1007/s10750-013-1524-7

[B25] Haas F (1969) Superfamilia Unionacea. Das Tierreich 88: 1–663.

[B26] Halnan CRE (1989) Cytogenetics of Animals. CAB International, Wallingford, 519 pp.

[B27] Hoeh WR Bogan AE Heard WH (2001) A phylogenetic perspective on the evolution of morphological and reproductive characteristics in the Unionoida. In: Bauer G Wächlter K (Eds) Ecology and Evolution of the Freshwater Mussels Unionoida. Springer-Verlag, Berlin, 257–280. doi: 10.1007/978-3-642-56869-5_14

[B28] Jara-Seguel P Peredo S Palma-Rojas C Parada E Lara G (2000) Quantitative karyotype of *Diplodon chilensis* (Gray, 1828) (Bivalvia: Hyriidae). Gayana (Zoologia) 64: 189–193.

[B29] Jenkinson JJ (1976) Chromosome numbers of some North American naiads (Bivalvia: Unionacea). Bulletin of the American Malacological Union, 16–17.

[B30] Jenkinson JJ (2014) Chromosomal Characteristics of North American and Other Naiades (Bivalvia: Unionida). Malacologia 57: 377–397. doi: 10.4002/040.057.0210

[B31] King M (1993) Species Evolution: the Role of Chromosome Change. Cambridge University Press, 336 pp.

[B32] Kolnicki RL (2000) Kinetochore reproduction in animal evolution: Cell biological explanation of karyotypic fission theory. Cell Biology 97: 9493–9497. doi: 10.1073/pnas.97.17.9493 10.1073/pnas.97.17.9493 PMC16892 10944218

[B33] Kongim B Panha S Naggs F (2006) Karyotype of land operculate snails of the genus *Cyclophorus* (Prosobranchia: Cyclophoridae) in Thailand. Invertebrate Reproduction and Development 49: 1–8. doi: 10.1080/07924259.2006.9652188

[B34] Kongim B Sutcharit C Tongkerd P Panha S (2009) Karyotype differentiation within the Elephant Pupinids Snail, *Pollicaria mouhoti* (Pfeiffer, 1862) (Caenogastropoda: Pupinidae). The Natural History Journal of Chulalongkorn University 9: 201–208.

[B35] Kongim B Sutcharit C Tongkerd P Tan SHA Quynh NX Naggs F Panha S (2010) Karyotype variation in the genus *Pollicaria* (Caenogastropoda: Pupinidae). Zoological Studies 49: 125–131.

[B36] Levan AR Fredga K Sandberg AA (1964) Nomenclature for centromeric position on chromosomes. Hereditas 52: 201–220. doi: 10.1111/j.1601-5223.1964.tb01953.x

[B37] Lillie FR (1901) The organization of the egg of *Unio*, based on a study of its maturation, fertilization and cleavage. Journal of Morphology 17: 227–292. doi: 10.1002/jmor.1050170204

[B38] Lopes-Lima M Teixeira EF Lopes A Varandas S Sousa R (2014) Biology and conservation of freshwater bivalves: past, present and future perspectives. Hydrobiologia 735: 1–13. doi: 10.1007/s10750-014-1902-9

[B39] Marshall BA Fenwick MC Ritchie PA (2014) New Zealand recent Hyriidae (Mollusca: Bivalvia: Unionida). Molluscan Research 34: 181–200. doi: 10.1080/13235818.2014.889591

[B40] McMichael DF Hiscock ID (1958) A monograph of freshwater mussels (Mollusca: Pelecypoda) of the Australian region. Australian Journal of Marine and Freshwater Research 9: 372–508. doi: 10.1071/MF9580372

[B41] Meesukko C (1996) Karyotype of freshwater amblemid mussels in Yom and Nan watersheds. Masters Thesis, Department of Biology, Faculty of Science, Chulalongkorn University, Bangkok, Thailand [In Thai with English Abstract]

[B42] Nadamitsu S Kanai T (1975) Chromosome of the freshwater pearl mussel *Margaritifera leavis* (Haas). Bulletin of Hiroshima Women’s University 10: 1–3.

[B43] Panha S (1990) The site survey and the study on reproductive cycles of freshwater pearl mussels in the Central Part of Thailand. Venus 49: 240–250.

[B44] Panha S (1992) Infection experiment of the glochidium of a freshwater pearl mussel, Hyriopsis (Limnoscapha) myersiana (Lea 1856). Venus 51: 303–314.

[B45] Panha S (1993a) Glochidiosis and juveniles production in a freshwater pearl mussel, *Chamberlainia hainesiana*. Invertebrate Reproduction and Development 24: 157–160. doi: 10.1080/07924259.1993.9672347

[B46] Panha S (1993b) All year breeding of *Physunio eximius* and *Scabies crispata* in the Mun River, Thailand. The Papustyla 7: 4–5.

[B47] Park GM Burch JB (1995) Karyotype analyses of six species of north America freshwater mussels (Bivalvia: Unionidae). Malacological Review 28: 43–61.

[B48] Patterson CM Burch JB (1978) Chromosomes of pulmonate mollusks. In: Fretter V Peake J (Eds) Pulmonates: Systematics and Ecology. Academic Press, New York, 171–217.

[B49] Petkevičiūtė R Stunžėnas V Stanevičiūtė G (2006) Polymorphism of the *Sphaerium corneum* (Bivalvia, Veneroida, Sphaeriidae) revealed by cytogenetics and sequence comparison. Biological Journal of the Linnean Society 89: 53–64. doi: 10.1111/j.1095-8312.2006.00657.x

[B50] Plouviez S Shank TM Faure B Daguin-Thiebaut C Viard F Lallier FH Jollivet D (2009) Comparative phylogeography among hydrothermal vent species along the East Pacific Rise reveals vicariant processes and population expansion in the South. Molecular Ecology 18: 3903–3917. doi: 10.1111/j.1365-294X.2009.04325.x 19709370 10.1111/j.1365-294X.2009.04325.x

[B51] Prié V Puillandre N (2014) Molecular phylogeny, taxonomy, and distribution of French *Unio* species (Bivalvia, Unionidae). Hydrobiologia. doi: 10.1007/s10750-013-1571-0 [published online 23 June 2013]

[B52] Rickart EA Mercier JA Heanney LR (1999) Cytogeography of Philippine bats (Mammalia: Chiroptera). Proceedings of the Biological Society of Washington 112: 453–459.

[B53] Roe KJ Hartfield PD Lydeard C (2001) Phylogeographic analysis of the threatened and endangered superconglutinate-producing mussels of the genus *Lampsilis* (Bivalvia, Unionidae). Molecular Ecology 10: 2225–2234. doi: 10.1046/j.1365-294X.2001.01361.x 11555264 10.1046/j.1365-294x.2001.01361.x

[B54] Rooney DE Czepulkowski BH (1992) Human Cytogenetics: A Practical Approach Vol. II. Malignancy and Acquired Abnormalities. 2nd ed Oxford University Press, New York, 293 pp.

[B55] Rosenberg G Davis GM Kuncio GS Harasewych MG (1994) Preliminary ribosomal RNA phylogeny of gastropod and unionoidean bivalve mollusks. Nautilus Supplement 2: 111–121.

[B56] Rosenberg G Tillier S Tillier A Kuncio GS Hanlon RT Masselot M Williams CJ (1997) Ribosomal RNA phylogeny of selected major clades in the Mollusca. Journal of Molluscan Studies 63: 301–309. doi: 10.1093/mollus/63.3.301

[B57] Sethi SA Selle AR Doyle MW Stanley EH Kitchel HE (2004) Responses of unionid mussels to dam removed in Koshkonong Creek, Wisconsin. Hydrobiologia 525: 157–165. doi: 10.1023/B:HYDR.0000038862.63229.56

[B58] Shan O Yufang A Xiaoping W Huiyin S (2001) Study on the karyotype of *Anodonta woodiana woodiana* (Bivalvia, Unionidae). Journal of Nanchang University (Natural Science) 25: 90–92.

[B59] Sumner AT (2003) Chromosomes: Organization and Function. Blackwell Publishing, London, 287 pp.

[B60] Sutcharit C Tongkerd P Kongim B Panha S (2013) A Handbook and the Photograph of Freshwater Mussels in Thailand. Chulalongkorn University, Bangkok, 12 pp [In Thai]

[B61] Thiriot-Quiévreux C Seapy R (1997) Chromosome studies of three families of pelagic heteropod molluscs (Atlantidae, Carinariidae and Pterotracheidae) from Hawaiian waters. Canadian Journal of Zoology 75: 237–244. doi: 10.1139/z97-030

[B62] Vannarattanarat S Zieritz A Kanchanaketu T Kovitvadhi U Kovitvadhi S Hongtrakul V (2014) Molecular identification of the economically important freshwater mussels (Mollusca: Bivalvia: Unionoida) of Thailand: developing species-specific markers from AFLPs. Animal Genetics 45: 235–239. doi: 10.1111/age.12115 24313464 10.1111/age.12115

[B63] Vaughn CC Tayler CM (1999) Impoundments and the decline of freshwater mussels: a case study of an extinction gradient. Conservation Biology 13: 912–920. doi: 10.1046/j.1523-1739.1999.97343.x

[B64] Vitturi R Rasotto MB Farrinella-Ferruzza N (1982) The chromosome number of 16 molluscan species. Bollettino di Zoologia 49: 61–71. doi: 10.1080/11250008209439373

[B65] Wang XJ Wang YJ Shi AJ Wang XZ (2000) Research on chromosomes of *Hyriopsis cumingi*. Sichuan Daxue Xuebo. Journal of Sichuan University 37: 252–256.

[B66] Whelan NV Geneva AJ Graf DL (2011) Molecular phylogenetic analysis of tropical freshwater mussels (Mollusca: Bivalvia: Unionoida) resolves the position of Coelatura and supports a monophyletic Unionidae. Molecular Phylogenetics and Evolution 61: 504–514. doi: 10.1016/j.ympev.2011.07.016 21827862 10.1016/j.ympev.2011.07.016

[B67] White MDJ (1978) Chain processes in chromosomal speciation. Systematic Zoology 27: 17–26. doi: 10.2307/2412880

[B68] Williams JD Warren ML Cummings KS Harris JL Neves RJ (1993) Conservation status of freshwater mussels of the United States and Canada. Fisheries 18: 6–22. doi: 10.1577/1548-8446(1993)018<0006:CSOFMO>2.0.CO;2

[B69] Woznicki P (2004) Chromosomes of the Chinese mussel *Anodonta woodiana* (Lea 1834) from the heated Konin Lakes system in Poland. Malacologia 46: 205–209.

[B70] Woznicki P Jankun M (2004) Chromosome study of *Anodonta anatina* (L., 1758) (Bivalvia, Unionidae). Folia Biologica (Kraków) 52: 171–174. doi: 10.3409/1734916044527593 10.3409/1734916044527593 19058556

[B71] Zhou D Zhou M Wu Z (1988) The karyotype of five species of freshwater snails of the family Viviparidae. Acta Zoologica Sinica 34: 364–370.

